# Chemoinformatics: Achievements and Challenges, a Personal View

**DOI:** 10.3390/molecules21020151

**Published:** 2016-01-27

**Authors:** Johann Gasteiger

**Affiliations:** Computer-Chemie-Centrum, University of Erlangen-Nuremberg, D-91052 Erlangen, Germany; johann.gasteiger@fau.de; Tel.: +49-89-795810

**Keywords:** chemoinformatics, chemical structure representation, chemical databases, data quality, inductive learning, data mining methods, property prediction, QSAR, QSPR, CASE, CASD

## Abstract

Chemoinformatics provides computer methods for learning from chemical data and for modeling tasks a chemist is facing. The field has evolved in the past 50 years and has substantially shaped how chemical research is performed by providing access to chemical information on a scale unattainable by traditional methods. Many physical, chemical and biological data have been predicted from structural data. For the early phases of drug design, methods have been developed that are used in all major pharmaceutical companies. However, all domains of chemistry can benefit from chemoinformatics methods; many areas that are not yet well developed, but could substantially gain from the use of chemoinformatics methods. The quality of data is of crucial importance for successful results. Computer-assisted structure elucidation and computer-assisted synthesis design have been attempted in the early years of chemoinformatics. Because of the importance of these fields to the chemist, new approaches should be made with better hardware and software techniques. Society’s concern about the impact of chemicals on human health and the environment could be met by the development of methods for toxicity prediction and risk assessment. In conjunction with bioinformatics, our understanding of the events in living organisms could be deepened and, thus, novel strategies for curing diseases developed. With so many challenging tasks awaiting solutions, the future is bright for chemoinformatics.

## 1. Introduction

From the very beginning, chemistry has derived most of its knowledge from observations and from data acquired through these observations. Only much later has theoretical chemistry matured to a point that, in some cases, it could make predictions accurate enough to satisfy chemical requirements. Nevertheless, many chemical phenomena are too complex to defy a treatment on first principles. Thus, the acquisition of chemical knowledge still relies to a large extent on experimental data; a process involving inductive learning: transforming data to information by combining related data and then information into knowledge by analyzing entire datasets. It had already been recognized 50 years ago that this process of inductive learning can benefit from using computer technology. Software has been developed that can process large amounts of data, more than a human researcher could do, and can do so very rapidly, again more than a scientist could achieve.

In the 1960s, several independent attempts were made in various fields of chemistry to use the power of computers to model and elucidate chemical phenomena. Foremost was the development of methods to store and retrieve chemical structure information [1,2]. Then, methods were developed to produce quantitative structure-activity/property relationships (QSAR/QSPR) for predicting physical, chemical, biological or environmental data of chemicals [3]. The development of mathematical methods to model such relationships led to the establishment of an entire scientific field: chemometrics [4]. It was also realized that cathode ray tubes offer the potential to graphically visualize three-dimensional molecular models. Several inroads were made to the prediction of chemical structure information from spectroscopic data (computer-assisted structure elucidation (CASE)) [5,6]. The DENDRAL project at Stanford University is considered as a seminal approach to the application of artificial intelligence to chemical problems [7]. In the late 1960s, several groups at Harvard, Brandeis, Stony Brook and the Technical University of Munich embarked on the development of systems for designing organic syntheses (computer-assisted synthesis design (CASD)) [8,9,10,11].

Diverse, and seemingly unrelated, as all of these studies initially were, it was realized more and more that the different developments had to struggle with similar problems, particularly with the representation, manipulation and retrieval of chemical structure information [12]. Furthermore, many of these approaches to develop computer methods for chemical applications were using the same mathematical methods for analyzing chemical data or for building quantitative models. Work on the various approaches over the next few decades increasingly showed that a field of its own was emerging at the interface of chemistry, computer science and mathematics. However, it was not until the late 1990s that a name was given to this field: chemoinformatics [13,14,15,16,17,18,19,20,21]. It has become clear that chemoinformatics can have applications in any field of chemistry and related sciences. We therefore adhere to the definition of chemoinformatics:

### Chemoinformatics is the Application of Informatics Methods to Solve Chemical

In this endeavor, chemoinformatics is using inductive learning, learning from data, for making predictions on chemical phenomena. Computational chemistry, on the other hand, is based on deductive learning by making use of a theory to make predictions.

Presently, chemoinformatics has found its most widely-accepted applications in the field of drug design. However, we want to emphasize that basically all fields of chemistry can benefit from chemoinformatics, as will be illustrated for some areas in the following discussion.

It should be emphasized that this paper does not attempt to provide a comprehensive review on chemoinformatics. A list of some additional leading references was provided by a reviewer and is integrated into the text. Rather, I want to give a personal view on some important achievements and interesting challenges that chemoinformatics could meet. This should serve to stimulate further research and development for understanding chemical phenomena.

## 2. Achievements

### 2.1. Databases

Clearly, the most widely-accepted achievement of chemoinformatics is that it provides access to chemical information in databases on a scale unattainable by working through the chemical literature. With presently 90 million known compounds, it would just be impossible to obtain an overview of the known chemistry without databases. Just to put the massive increase in chemical information in recent decades into perspective: when the present author obtained his PhD in chemistry, only 1.5 million compounds were known. Most chemical researchers take access to chemical information through databases so much for granted that they do not appreciate that databases on chemical information would not exist without research in chemoinformatics that laid the foundation for such databases. Furthermore, chemists can communicate with databases in their international language, the graphical language of structure diagrams and reaction equations.

Many methods had to be developed for allowing the construction of databases on chemical information:

- graphical input of chemical structures
- graphical output of chemical structures
- unique and unambiguous representation of chemical structures
- conversion of names to structures and *vice versa*
- ring perception
- aromaticity perception
- full-structure search
- sub-structure search
- similarity search
- development of file formats for information exchange [22,23,24,25,26]

All this runs in the background without the user being aware of the technology involved. However, chemoinformaticians first had to develop those methodologies. Several publications provide an overview of databases in use in chemistry [27,28,29,30].

Suffice it to say that modern chemical research would not be possible without the developments in chemoinformatics incorporated into databases.

The availability of chemical data in databases provides a good basis for the development of models. Thus, it was shown with the development of the CORINA system that the information on 3D structures of 240,000 compounds in the Cambridge Crystallographic Database could be harnessed for the prediction of the 3D structure of any organic molecule [31,32]. In effect, information on 3% of molecules allowed the prediction of the 3D structure of more than 99.5% of all organic molecules.

### 2.2. Property Prediction (QSAR/QSPR)

**Figure 1:**
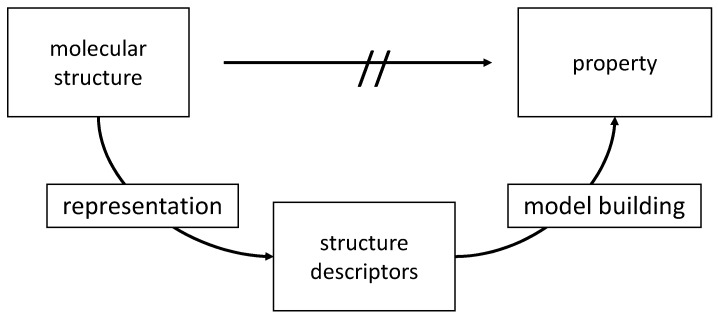
The basic approach to QSAR/QSPR: In the first step, a molecular structure is represented by structure descriptors. In the second step, a dataset of structures as represented by their descriptors and their associated properties is submitted to a data analysis and model building method.

The relationships between many chemical and, particularly, biological data of compounds and their structure are too complex to be directly predicted on first principles. In such cases, an indirect two-step process has to be invoked (Figure 1).

First, structure descriptors have to be derived for the structures of a dataset. Then, a model for the relationship between the structure descriptors and the investigated property has to be established by a data analysis or model building technique. For both steps, a large amount of methods have been developed. A host of methods, counting in the thousands, for calculating structure descriptors, is available [33]. More and more attention now shifts to the use of structure descriptors that can be interpreted and thus provide a model that increases insights into the relationship between the structure of a compound and its properties. A clear classification of molecular descriptors in describing the 1D, 2D, 3D structure or the molecular surface properties is quite helpful in this endeavor [34]. Furthermore, increasing attention is given to the representation of chemical compounds that goes beyond a molecular characterization [35].

For data analysis and model building, quite a variety of mathematical methods have been developed that come under different notations:

- statistical methods
- chemometrics
- pattern recognition
- data mining
- machine learning
- neural networks
- genetic algorithms
- variable selection algorithms
- definition of applicability domain

A large number of physical, chemical and biological properties of chemical compounds have been modeled and predicted by this two-step approach. In realizing that a QSAR/QSPR approach is strongly dependent on the set of descriptors selected and the modeling technique chosen, sometimes, several different models have simultaneously been developed. This is certainly quite recommendable in order to learn the strengths and pitfalls of the various descriptor sets and modeling techniques. However, to use these different results to coin a consensus model is highly questionable as it tries to average results by voting, a method that is quite unusual in science. Rules for developing quality QSAR models have been defined [36,37]. An extensive overview indicating achievements and problems with QSAR/QSPR with 300 references has appeared [38].

### 2.3. Drug Design

By far the largest number of applications of chemoinformatics has been made in drug design. Methods have been developed for:

- lead discovery (both ligand- and structure-based methods)
- lead optimization
- modeling of ADMET properties (adsorption, distribution, metabolism, excretion and toxicity)

A host of publications can be found in the literature. Some overviews on chemoinformatics tools and on ADME models can be found in refs [39,40,41].

Chemoinformatics has made substantial contributions to the development of a variety of new drugs. This approach has matured to a point that all major drug companies have a chemoinformatics department, and practically all drugs that have newly been developed have involved in one or another step chemoinformatics methods.

### 2.4. Analytical Chemistry

Most tasks in analytical chemistry involve a classification problem, such as the assignment of a sample to a specific category. It was recognized quite early on that the classification of analytical samples can greatly benefit from computational methods. This resulted in the development of the field of chemometrics; already in the mid-1980s, specialized journals for this field were conceived of and still thrive. This field found its own name way before the name chemoinformatics appeared. However, it is quite clear that chemometrics should be considered as a subfield of chemoinformatics. Many of the data representation techniques and the mathematical methods for data analysis are used in both domains.

## 3. Challenges

### 3.1. Three Fundamental Questions of A Chemist

Academic research is concerned with increasing our knowledge, while industrial applications aim at producing new compounds or materials with desired properties. Chemoinformatics can assist chemists for both tasks in their daily work.

In academia, mostly QSAR and QSPR studies are performed that can provide insights into the relationships between structure and properties, particularly so if structure descriptors are used that can be interpreted and data analysis methods are employed that indicate the amount of contribution of the various descriptors.

However, as said, the aim of industry is to produce compounds with new or better properties, be it a drug, a paint, a plastic, *etc.* This goal was quite elegantly emphasized by George S. Hammond in his Norris Award Lecture in 1968:

The fundamental and lasting objective of synthesis is not production of new compounds but production of properties.

If this is accepted, chemists have to face three fundamental questions.

- Which compound will have the desired property?
- How can I make this compound?
- Did I make this compound (what is the product of my reaction)?

Additionally, all three areas can benefit from the use of chemoinformatics methods:

- structure-property relationships (QSPR/QSAR)
- synthesis design (CASD)
- prediction of the outcome of a reaction and structure elucidation (CASE)

The areas of QSAR and QSPR are quite well developed. However, a shift in interest from models to interpretation can clearly be discerned. Structure descriptors and data analysis methods that provide insight and allow an interpretation have to be chosen.

Whereas much work has been done in QSPR, chemoinformatics methods for the other tasks are still in their infancy or not well accepted by chemists. Much more efforts should be put into the development of methods for computer-assisted synthesis design, computer-assisted structure elucidation and the prediction of the course and outcome of chemical reactions given the importance of these tasks.

### 3.2. Toxicity Prediction and Risk Assessment

Society has become increasingly interested and concerned about the impact of chemicals on the environment and on human health. Therefore, chemicals should be introduced into the market or used only if they have been proven to be safe. As a consequence, legislation has been introduced in Europe to ensure the safety of chemicals that are used in substantial amounts. The REACH (Registration, Evaluation, Authorization and Restriction of Chemicals) initiative was put into law in 2007 by the European Union to ensure that enough information on potential negative impacts on human health of high-production chemicals becomes available [42]. As this legislation includes both the manufacturing and the importing of chemicals, it has impact not only in the European Union, but worldwide. Other countries such as Canada, China, Japan and Korea have introduced similar legislation. To limit vertebrae animal testing in this registration process, computational toxicity models can offer important applications for chemoinformatics.

In the same framework of mind, the European Union has put into law in 2003 the 7th Amendment to the Cosmetics Directive that prohibits from 2009 on animal testing of cosmetics ingredients [43]. Again, this offers interesting applications for computational toxicity models as alternatives to animal testing. Both the REACH initiative and the Cosmetics Directive will provide a host of data for the development of better computational toxicity models. With more and more data becoming available, we will be able to increase our knowledge about the relationships between chemical structure and toxicity.

Furthermore, the impact of chemicals on the environment is of much concern in society. Models for persistence, bioaccumulation and toxicity of chemicals in all kinds of organisms are asked for.

### 3.3. Modeling Biological Systems

The next step is then a focus on unraveling the events in living organisms. It should be realized that life is maintained by (bio)chemical reactions; modeling and understanding them is essential for getting deeper insights into the events that keep living species alive. This could provide a basis for curing diseases. New research fields have been conceived of, with names such as systems chemistry, systems biology and systems chemical biology. Whatever the names, the aim is to further an understanding of biological systems even to a point that they can be altered. A collaboration between chemoinformatics and bioinformatics is essential for this purpose.

The newest developments aim at modeling entire human organs. Large-scale government-supported projects in the U.S. and in Germany have been initiated to develop models for the human liver, the virtual liver v-Liver at EPA [44] and the German virtual liver projectnetworks [45].

## 4. Tasks and Inroads

The previous section dealt with challenges on a more global basis. In this section, we will deal with more specific tasks that have to be solved to face the global challenges. Clearly, we can highlight only a few of these tasks. In order to emphasize that inroads to some of these tasks have already been made, occasionally some results will be mentioned for illustration.

### 4.1. Access to Data

**Figure 2:**
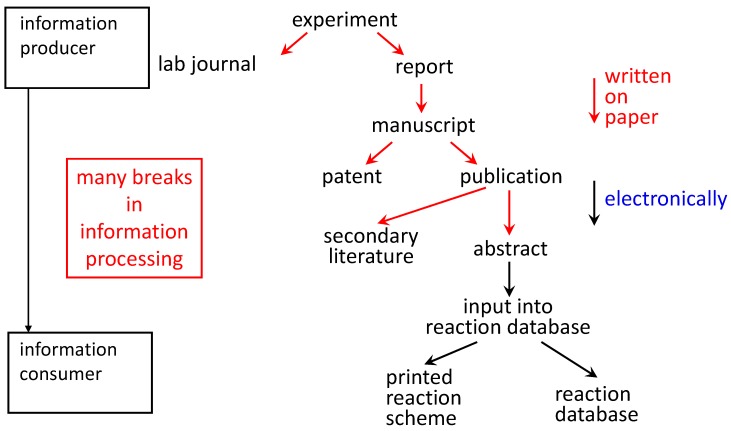
At present, the path of information from the experiment to end up in a database involves many steps of manual intervention where the information is newly conceived.

Chemoinformatics critically relies on data for the development of models. The quality of data is therefore of crucial influence on the quality of a model. It is sad to say that many chemical and particularly biological datasets are full of errors (some estimates are in the 20% range!). Thus, improving the quality of data is essential for the further development of the field of chemoinformatics and its power for increasing our knowledge of chemistry [46,47]. Methods for eliminating errors from datasets and for making quality checks before data are stored in a database have to be developed. All information that is available on a compound (all properties, all spectra) should go into a database. Databases on chemical reactions are notoriously incomplete. Information has to be provided on the full stoichiometry, on side products and their amount and on all reaction conditions (ratio of starting material, solvent, reaction time and temperature). Only then can we better learn about chemical reactivity and the course of chemical reactions. Presently, there are many manual interventions on the path from providing information on an experiment until it finally ends up in a database (Figure 2). All of these manual interventions involve the danger that errors are introduced or information is dropped.

**Figure 3:**
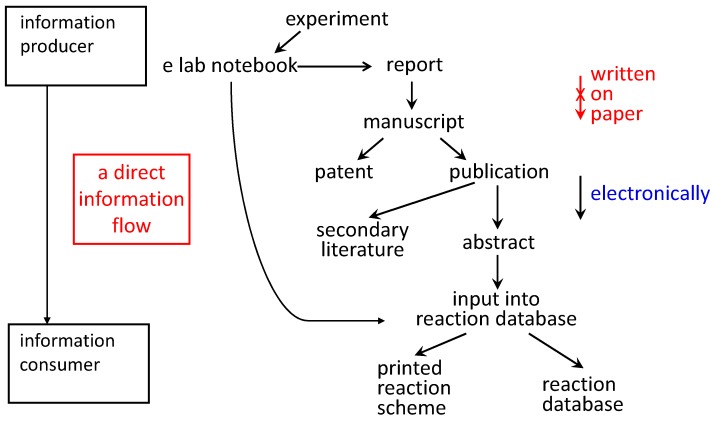
When the experimental observations are written into an electronic laboratory notebook, the information can flow into a variety of different information products without it being rewritten.

The use of electronic laboratory notebooks could lay the foundation for a direct flow of information from the information producer (the experimenter: who knows the information best) to the information consumer accessing a database (Figure 3). This will prevent errors being introduced into the information products. Clearly, formats for the storing of information should be used that have semantics and allow it to be parsed for further computer processing. Work on the Chemical Markup language is instrumental for that purpose [48,49,50,51].

It should be emphasized that reaction information is used only for illustration. The same arguments apply to any type of chemical information.

Many data are produced, and hidden, in industry. If these data were released, academia could use them for building models and, thus, substantially increase our insight into chemistry. Clearly, some data are of crucial interest to industry and will therefore have to stay in-house. However, many data are from projects that have been abandoned or are no longer of crucial interest. For academia, such data could be a treasure trove.

### 4.2. QSPR/QSAR

It has already been said that many thousands of different methods for calculating structure descriptors are available. However, most of them do not easily lend themselves to interpretation. On the other hand, the trend in QSAR/QSPR certainly moves away from building models that might be good in predicting data to models that can be interpreted. This requires the use of structure descriptors that can be interpreted and the use of data modeling techniques that show a direct relationship between a descriptor and the weight with which the descriptor enters into the model.

It can also be envisaged that chemoinformatics and theoretical chemistry will collaborate more in the future. In fact, it has already been shown that data calculated by high-level quantum-mechanical methods can be modeled by structure descriptors that can rapidly be calculated by a QSPR approach [52].

### 4.3. Drug Design

Most applications of chemoinformatics are made in the field of drug design. Considering the importance of this field and the power of chemoinformatics methods, these applications will rightly continue to thrive. Clearly, there are certainly problems that need new ideas and new methods. The conformational flexibility of ligands and, particularly, of proteins still poses problems that deserve continuing attention. New global approaches to drug design covered by names like chemogenomics or pharmacogenomics have been introduced, fields that ask for the collaboration of chemoinformatics with bioinformatics. Uncovering the mechanistic merits of traditional medicines, such as Traditional Chinese Medicine, and merging these with the framework of Western-style medicine promises to lead to new treatments.

### 4.4. Analytical Chemistry

Analytical chemistry provides a host of problems to be studied by chemoinformatics methods. Often, such studies are listed under the name of chemometrics. Chemometrics is characterized by usually representing the chemical objects by measured data and analyzing these data by the same kind of mathematical methods as employed in QSAR/QSPR studies. Thus, chemometrics should be considered as a subfield of chemoinformatics. Chemoinformatics encompasses a much wider range of investigations, particularly also those that characterize chemical structures and reactions.

**Figure 4:**
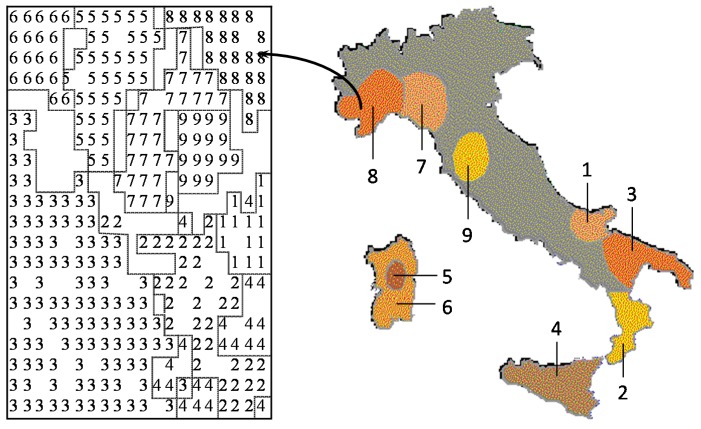
Comparison of the map obtained by a self-organizing neural network for olive oils from nine Italian olive growing areas with the map of Italy.

Many studies in analytical chemistry deal with a classification problem, assigning the chemical objects to a certain class. It is our strong belief that any study should first be done with an unsupervised learning method, such as a principal component analysis or a self-organizing neural network. The unsupervised learning method uses only the set of descriptors; the property to be modeled is not included in the training phase. By using different sets of descriptors for characterizing the objects, that set has to be found that separates the objects best. With this set, then, a predictive model should be built by including the property to be studied by a supervised learning method. Using an unsupervised learning method may allow the discovery of interesting information not directly sought, but hidden in the data. This point, as well as the relationship between chemometrics and chemoinformatics will be illustrated by a study on the classification of Italian olive oils. Each olive oil sample was characterized by its content of six different fatty acids. Several different studies had been performed on this dataset and published in chemometrics journals, which could all quite well be taken as chemoinformatics investigations. In a further study, by using a self-organizing neural network, a two-dimensional map was produced that allowed the separation of the samples into the various oil-growing regions of Italy with 97% accuracy. However, as an additional benefit, it was found that the classification map also reflected the geographic map of Italy (Figure 4) [53]. Apparently, the information on the regional influences on olive oil composition was hidden in the data.

**Figure 5:**
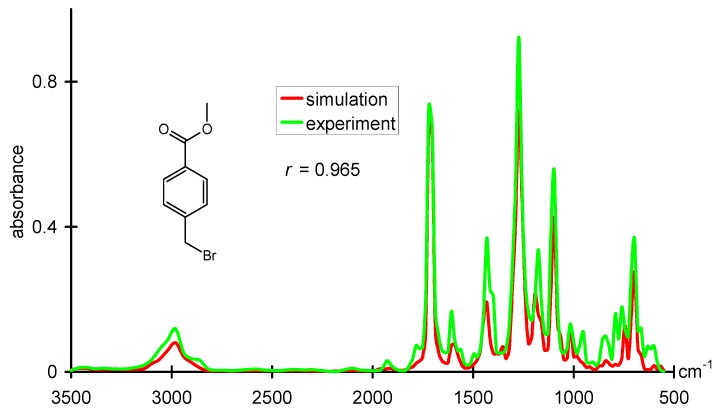
Comparison of an experimental infrared spectrum with an infrared spectrum obtained from modeling a dataset where structures are represented by a radial distribution function.

Computer-assisted structure elucidation (CASE) was one of the fields where chemoinformatics methods were introduced in the 1960s. Much work was done by several groups in the following decades. However, no comprehensive CASE system could be achieved that was widely used. On the other hand, chemists spend a lot of time deciphering the structure of an unknown compound from spectral data. Therefore, assisting a chemist in this process by a CASE system would be of much merit. Modeling the relationships between the structure of a compound and its spectral data is a prerequisite for a CASE system. For ^13^C- and ^1^H-NMR data, quite good correlations have already been developed. For infrared spectra, only the bands for bond vibrations are traditionally assigned. A large part of the spectrum in the fingerprint region defied a direct interpretation by correlation methods. However, with a novel structure representation, a radial distribution function code that is able to encode the entire 3D structure, excellent predictions of infrared spectra could be made. Figure 5 gives an example for such a prediction and compares it to the experimental IR spectrum [54]. The approach used, a counterpropagation neural network, even allowed a reverse application, the prediction of the 3D structure of an organic compound from its infrared spectrum [55].

In the meantime, the calculation of infrared spectra by quantum mechanical methods has matured to a point that compares well with the experimental spectrum. This is an area where inductive learning by chemoinformatics methods meets deductive learning by theoretical methods.

Although much work has been done on the prediction of mass spectra from the structure of a compound, no general convincing approach is yet available. Particular difficulties arise from the strong dependence of a mass spectrum on the special technique applied. However, as mass spectra have great importance for structure determination, still more work should be done for the prediction of mass spectra from structural data. A CASE system would need a structure generator in addition to prediction methods for the various spectral data. It is hoped that work in this area is again taken up and intensified.

### 4.5. Organic Chemistry

Work on synthesis design systems was one of the roots of chemoinformatics in the 1960s. In spite of the large amount of work devoted to this field, no single system has found wide-spread use. The reasons are manifold: complexity of the problem, low efficiency of computers at that time, unfamiliarity of organic chemists with computers. Furthermore, organic chemists hold the design of a synthesis as one of the most interesting tasks of their profession, and therefore, they do not want to delegate this to a computer. However, the statement made by Herb Gelernter in 1973 (private communication) is still valid: “The amount of information to be processed and the decisions between many alternatives suggests the use of computers in organic synthesis design.”

In order to come up with a synthesis that is as efficient and economical as possible, a large amount of information and knowledge is required, a requisite that might not always be best available to the chemist. There, information in databases on availability and prizes of starting materials, on toxicity and dangerous properties of intermediates, as well as chemoinformatics models on chemical reactivity, knowledge on short and efficient synthesis strategies could certainly help in coming up with superior syntheses. All of these criteria would have to be considered in a CASD system and will have to be balanced against each other. As the balancing of these criteria is often strongly dependent on the specific interests of a chemist, it is desirable to develop CASD systems that a chemist can directly interact with and thus determine the course of a designed synthesis. In other words, let us bring together the best of the two, the chemist and the computer, in a team: the computer storing a lot of information and tirelessly exploring many alternatives; the chemist using his or her training and knowledge and his or her ability of lateral thinking.

In fact, it can be observed that interest in making the design of a synthesis more efficient has caught up with organic chemists and with process chemists. Thus, recently, the complexity of organic molecules was defined and was considered dependent on the current technology of synthesis [56]. Various synthesis schemes were compared by this measure, showing the superiority of recently-performed syntheses of complex molecules as against syntheses performed some time ago. This work followed up on prior studies on the definition of the synthetic availability of compounds [57]. Both studies compared the computed values with estimates made by organic chemists [58].

The other highly important topic for an organic chemist is the prediction of chemical reactivity and of the course of chemical reactions, as was emphasized to be one of the three fundamental questions of a chemist (see Section 3.1). A major step forward in these areas can only be made when better databases on chemical reactions become available. The best would certainly be if kinetic data on chemical reactions were obtainable. In the future, chemoinformatics and theoretical chemistry should join efforts to model chemical reactivity.

### 4.6. Toxicology

The REACH legislation and the Cosmetics Directive have emphasized and focused interest on computational models for toxicity prediction and risk assessment of chemicals. Many studies are being performed and made available; a few leading references are given as refs [59,60,61,62,63]. It is quite clear that there cannot be a universal model for the relationship between a chemical structure and its “toxicity”. Rather, separate models have to be developed for individual toxicity endpoints. Therefore, the first step in modeling toxicity has to be the classification of a chemical according to its toxic mode-of-action [64]. The entire field suffers from a lack of high quality data, and many models are therefore of doubtful usefulness. The definition of the applicability domain of a toxicity model is essential for any study. The release of data from industry could provide a major step forward. In this context, the approach taken by the Environmental Protection Agency of USA is to be commended. In their ToxCast and Tox21programs, they measured a set of chemicals against a battery of more than 100 *in vitro* cell-based assays [65]. These data are made available to the scientific community for the development of predictive models. Quite a few such models have been developed at EPA [66,67,68,69,70,71].

### 4.7. Biochemistry

**Figure 6:**
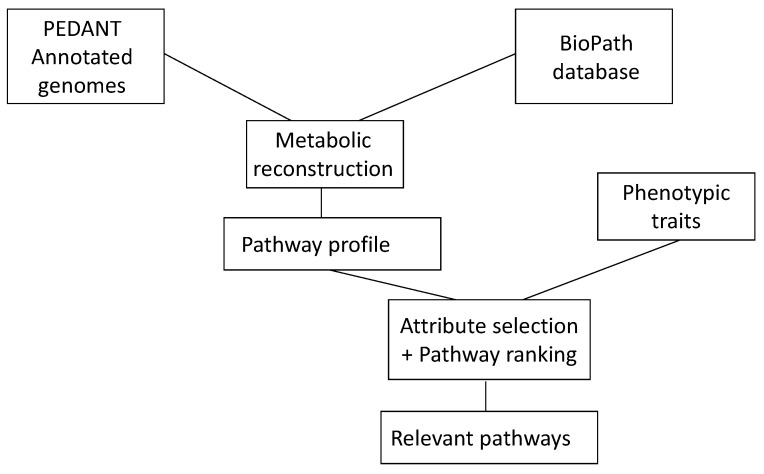
The combination of genetic information as contained in the database PEDANT with information on biochemical reactions as contained in the BioPath database allows one to determine the relevant pathways of a disease.

An understanding of the events in living systems, and to a large part, they are (bio)chemical reactions, is of much current interest. To make progress in this endeavor, a collaboration between chemoinformatics and bioinformatics is essential. Chemoinformatics usually deals with small molecules and their reactions; bioinformatics starts with genes and how they express proteins. Both approaches meet in the investigation of enzymes and their catalysis of biochemical reactions. Providing information from both chemoinformatics and bioinformatics in databases is essential for any study in this field. It was shown how a database of biochemical reactions in conjunction with a database of genomes of microorganisms could be used to uncover metabolic pathways relevant to phenotypic traits of microbial genomes in diseases (Figure 6) [72].

In another study, a chemical systems biology approach of reverse pathway engineering was used in the prediction of bacterial flavor-forming pathways [73].

### 4.8. Biology

The application of chemoinformatics methods is not limited to chemistry. All related scientific fields that are somehow dealing with chemical information are prone to the applications of chemoinformatics methods. As an example from botany, the classification of the family of Asteraceae plants based on an analysis of their secondary metabolites is mentioned here [74].

## 5. Summary

Chemoinformatics has developed from scattered individual beginnings to a full-fledged scientific discipline. Many problems have been solved, and interesting results were obtained. Databases on chemical information are probably the most visible achievements. Chemical research could not be pursued on the present high level without access to databases on chemical information. Thus, chemoinformatics has substantially changed how research in chemistry is being performed.

A methodology has been developed for the prediction of properties that cannot directly be calculated by theoretical methods. Thus, many physical, chemical or biological properties have been predicted from information on the structure of a compound.

Chemoinformatics has found many applications in drug design. All major pharmaceutical companies have a chemoinformatics department, and practically all new drugs were developed with some involvement of chemoinformatics methods.

However, chemoinformatics could find potential applications in any field of chemistry or related sciences. These areas are much less developed. Work in these fields could reap many benefits and interesting results. Many data that are presently used for model building are of doubtful accuracy or contain errors. Access to high quality data is of crucial importance.

Society is quite concerned about the influence of chemicals on human health and the environment. Chemoinformatics methods could assist in the prediction of toxicity and the assessment of the risk of chemicals and, thus, help to reduce animal testing.

Modeling the events in living organisms has recently caught much interest in order to develop a better understanding of diseases and their curing. As these processes involve many biochemical reactions, chemoinformatics methods are asked for, best in collaboration with bioinformatics methods.

With all of these challenges, chemoinformatics has a bright future; it is an interesting field to work in and is embracing scientists that have new ideas. The importance of computational methods in chemistry has been emphasized in the press release of the Nobel Prize in Chemistry 2013: “Today the computer is just as important a tool for chemists as the test tube.”
